# Investigating differences in trunk muscle activity in non-specific chronic low back pain subgroups and no-low back pain controls during functional tasks: a case-control study

**DOI:** 10.1186/s12891-019-2843-2

**Published:** 2019-10-22

**Authors:** Rebecca Hemming, Liba Sheeran, Robert van Deursen, Valerie Sparkes

**Affiliations:** 1 Arthritis Research UK Biomechanics and Bioengineering Centre, School of Healthcare Sciences, Cardiff University, 13.20 Eastgate House, 35-43 Newport Road, Cardiff, Wales CF24 0AB UK; 2 Arthritis Research UK Biomechanics and Bioengineering Centre, School of Healthcare Sciences, Cardiff University, 2F25A Cardigan House, Heath Campus, Cardiff, Wales CF14 4XN UK

**Keywords:** Non-specific chronic low back pain, NSCLBP, Muscle activity, Trunk, Functional movement

## Abstract

**Background:**

Trunk muscle dysfunction is often regarded as a key feature of non-specific chronic low back pain (NSCLBP) despite being poorly understood and variable with increases, decreases and no change in muscle activity reported. Differences in thoraco-lumbar kinematics have been observed in motor control impairment NSCLBP subgroups (Flexion Pattern, Active Extension Pattern) during static postures and dynamic activities. However, potential differences in muscle activity during functional tasks has not been established in these subgroups to date.

**Methods:**

A case-control study design recruited 50 NSCLBP subjects (27 Flexion Pattern, 23 Active Extension Pattern) and 28 healthy individuals. Surface electromyography determined muscle activity during functional tasks: reaching upwards, step-down, step-up, lifting and replacing a box, stand-to-sit, sit-to-stand, bending to retrieve (and returning from retrieving) a pen from the floor. Normalised (% sub-maximal voluntary contraction) mean amplitude electromyography of bilateral musculature (transversus abdominis/internal oblique, external oblique, superficial lumbar multifidus and longissimus thoracis) was analysed using Kruskal-Wallis and post-hoc Mann-Whitney U tests.

**Results:**

Transversus abdominis/internal oblique activity was significantly increased in the Flexion Pattern group compared to controls during stand-to-sit (*p* = 0.009) on the left side only. External oblique activity was significantly greater in the Active Extension Pattern group compared to controls during box lift (*p* = 0.016) on the right side only. Significantly greater activity was identified in the right Superficial lumbar multifidus during step up (*p* = 0.029), reach up (*p* = 0.013) and box replace (*p* = 0.007) in the Active Extension Pattern group compared to controls. However left-sided superficial lumbar multifidus activity was significantly greater in the Flexion Pattern group (compared to controls) only during stand-to-sit (*p* = 0.009). No significant differences were observed in longissimus thoracis activity bilaterally during any task. No significant differences between NSCLBP subgroups were observed.

**Conclusions:**

Muscle activity in these NSCLBP subgroups appears to be highly variable during functional tasks with no clear pattern of activity identified. The findings reflect inconsistencies and variability in trunk muscle activity previously observed in these NSCLBP subgroups. Further work evaluating ratios of muscle activity and changes in muscle activity throughout task duration is warranted.

## Background

Non-specific chronic low back pain (NSCLBP) is highly complex with a multitude of physical, cognitive and lifestyle factors contributing to the disorder. Often the dominant driver for pain may be linked to movement and posture behaviour indicating a mechanical basis for the disorder [1]. People with NSCLBP have been found to demonstrate differences in temporal and spatial parameters of trunk muscle activity, although the nature of these differences is poorly understood with substantial variability reported [2, 3].

This variability may be due to lack of definition of homogeneous NSCLBP subgroups; therefore, identifying specific NSCLBP subgroups using validated subclassification approaches is paramount [4]. One such multidimensional classification system (MDCS) which categorises the disorder into movement impairment and/or motor control impairment (MCI) subgroups has been proposed [1]. Patients within these subgroups display high levels of fear-avoidance, and adopt subgroup-specific maladaptive postures and movement strategies, potentially exacerbating the disorder [5–7]. It is proposed that NSCLBP individuals with flexion pattern MCI (FP) demonstrate an inability to activate lumbar multifidus (LM), reporting pain during flexion biased activities, whereas extension pattern MCI (AEP) individuals may present with hyperextended lower lumbar postures with dominant erector spinae and LM activity to actively ‘hold’ themselves in lumbar hyperextension and report pain during more extended/upright) activitiesO’Sullivan [1].

Previous work evaluating trunk muscle activity in MDCS subgroups in sitting demonstrated AEP individuals having spinal musculature hyperactivity compared to healthy and FP groups [6, 7]. Similarly, increased abdominal muscle activity (EO and TrIO) in FP and AEP subgroups, compared with healthy individuals, during repositioning tasks in sitting and standing is reported [8].

This suggests that muscle hyperactivity is prevalent in the presence of pain, but differences in muscle activity between NSCLBP subgroups may not be sufficient to provide a rationale for differential diagnosis. Reasons for this may be due to static postures not being sufficiently challenging for the trunk muscles. We hypothesise that different subgroups of NSCLBP would accomplish more challenging functional tasks differently. To date this hypothesis has not been tested in functional tasks which could be considered more relevant than static postures.

This study investigated trunk muscle activity in subgroups of NSCLBP patients and healthy individuals during functional tasks, to identify whether differences in muscle activity are task dependent or whether a consistent pattern of muscle activity is identified irrespective of task performed.

This will inform an understanding of subgroup trunk muscle dysfunction, which may influence spinal loading and pain [6, 7], thus enabling refinement of motor control approaches for specific subgroups of NSCLBP patients [9].

The hypothesis for this case-control study is that differences in trunk muscle activity between NSCLBP subgroups and healthy individuals will be observed during a series of functional tasks reflecting a range of flexion-dominant and extension-dominant trunk motions.

## Methods

NSCLBP patients were recruited from physiotherapy waiting lists in Cardiff and Vale University Health Board, Cardiff, UK. Fifty NSCLBP and 28 healthy individuals volunteered. Sample size calculation is reported elsewhere [10]. Power set at priori at 0.7, alpha level of 0.05, a sample of 24 subjects per group was calculated.

Inclusion and exclusion criteria for NSCLBP and healthy individuals are outlined in Tables 1 and 2. NSCLBP subjects were classified independently by two physiotherapists (RH, LS) based on MDCS criteria [1]. Only subjects classified as FP or AEP (both clinicians in agreement) were included. To establish NSCLBP classification a comprehensive subjective and objective assessment was conducted. Full assessment procedures are published elsewhere [1, 11]. Gender, age, anthropometric data (weight, height) were collected. Patient reported measures for pain (Visual Analogue Scale (VAS)) [12], disability (Oswestry Disability Questionnaire (ODQ)) [13], psychological distress (Distress and Risk Assessment Method (DRAM)) [14] and fear of movement (Tampa Scale of Kinesiophobia (TSK)) [15] evaluated baseline characteristics. Data collection was conducted at the Research Centre for Clinical Kinesiology, Cardiff University.

**Table 1:** Inclusion and exclusion criteria for the non-specific chronic low back pain (NSCLBP) group

Inclusion criteria for the NSCLBP group	Exclusion criteria for the NSCLBP group
• Aged 18–65 years • History of chronic LBP (> 12 weeks) • Pain in the lumbar and/or buttock region (defined as pain reported below the level of T12 and no lower than the buttock line) • Clear mechanical basis of the disorder aligned with specific aggravating and easing postures and movements, with distinct symptom relief observed during movement conducted in the opposing direction of reported pain provocation (assessed subjectively and objectively) • Clinical diagnosis of specific MCI - either FP or AEP	• Signs of serious spinal pathology (Red flags) including significant trauma, unexplained weight loss and widespread neurologic changes • Any vestibular, visual or neurological dysfunction affecting balance • Current radiating symptoms (and/or neurological deficit) below the level of the buttock crease • Current pregnancy or breastfeeding • History of spinal surgery, fracture or malignancy • Inability to perform any of the functional tasks unaided • Inability to read written English language documents and follow verbal instructions in English • Not fulfilling the inclusion criteria

**Table 2:** Inclusion and exclusion criteria for the healthy group

Inclusion criteria for the healthy group	Exclusion criteria for the healthy group
• Aged 18–65 years	• History of LBP or any lower limb pain in the last 2 years • Any vestibular, visual or neurological dysfunction affecting balance • Current pregnancy or breastfeeding • History of spinal surgery, fracture or malignancy • Previous LBP with symptoms radiating below the level of the buttocks • Inability to complete the tasks required • Inability to read written English language documents and follow verbal instructions in English

### Data collection

Surface Electromyography (sEMG) data was collected through an 8 Channel Bortec EMG system (Octopus Cable Telemetric System, Bortec Electronics Inc., Calgary, Canada), synced with Vicon® Nexus software (Nexus 1.8.2 Vicon Motion Systems, Oxford, UK). sEMG has been shown to be a reliable tool in the assessment of paraspinal muscle in both healthy individuals and LBP patients [16]. The sEMG battery pack was linked to the main amplifier through a single fixed fibre-optic cable. Snap electrode leads were attached to each electrode [17].

Prior to sEMG electrode placement the skin was prepared through shaving and cleaning with alcohol wipes [18] and skin impedance tested (satisfactory if < 10 kΩ) [19]. Disposable, self-adhesive Ag/AgCl dual snap electrodes (Noraxon, Arizona, USA) with 1cm^2^ circular conductive areas and 2 cm inter-electrode distance were placed parallel to the muscle fibres of superficial Lumbar Multifidus (sLM), Longissimus Thoracis (LT), Transversus Abdominis/Internal Oblique (TrA/IO) and External Oblique (EO) muscles bilaterally, as per SENIAM guidelines [18]. An earth electrode was placed over the left iliac crest. All electrode placement was conducted by an experienced musculoskeletal physiotherapist.

Differential pre-amplifiers with fixed gain of 500, input impedance of 1OGOhm, common rejection ratio set at 115 dB and a sampling frequency of 10 Hz to 1000 Hz were used [8, 17]. Visual inspection of the sEMG data was conducted via an oscilloscope view within Vicon®.

sEMG data was normalised to sub-maximal voluntary contractions (SMVC). A crook-lying double leg raise was used to achieve SMVC of the abdominal muscles and a prone lying double knee lift for the LT and sLM muscles [17]. Three SMVCs were recorded over 3 s with a 30 s rest between trials [17].

Nine functional tasks were evaluated (reach up, sitting-to-standing, standing-to-sitting, step up, step down, box lift, box replace, bending to retrieve and returning from retrieving a pen from the floor). These reflected a range of functional activities and a selection of flexion-related (e.g. bend to retrieve) and extension-related (e.g. reaching upwards) tasks to potentially stress the direction of pain provocation hypothesised to be present in each MCI. Each task was repeated and recorded three times which is reflective of previous study protocols identifying regional spinal differences in back pain populations in functional activities [20–22].

### Data processing and analysis

Raw signals were full-wave rectified and band pass filtered (zero phase lag, 20 Hz cut-off frequency) using 2nd order, bidirectional Butterworth filter resulting in a linear envelope for each channel [18] using a custom-developed MATLAB routine. The signal was amplified further by a gain of 2000 using a 20 Hz high pass filter to suppress any potential movement artefacts. Data was visually inspected through graphical representation in MATLAB (version R2013a). Mean amplitude sEMG was calculated for the duration of the functional task (see supplementary material for details). The start and end of each task were established through synchronisation with kinematic data collected using a Vicon® motion analysis system (details published elsewhere) [10]. Where any anomalies in the data were apparent, the raw sEMG was identified and omitted from final data analysis. Data were exported to Excel and imported into SPSS for analysis (version 20.0, IBM Corp, Armonk, NY, USA). Normalised amplitude sEMG (%) was calculated as: (processed sEMG / SMVC)*100 across each functional task.

Full details of the electrode placement, SMVC procedures, standardisations for task performance and sections of the tasks used for sEMG analysis are included in an additional file (see Additional file 1).

### Statistical analysis

Statistical analysis were performed according to normal distribution and homogeneity of variance of the %SMVC data [23]. Differences in demographic characteristics and questionnaires between FP, AEP and healthy groups were determined using: one-way ANOVA with post-hoc comparisons (Bonferroni) for age and height; independent samples Kruskal-Wallis for mass and BMI and Chi^2^ for gender. ODQ and TSK scores were summed and averaged across each group to be regarded as interval-ratio data (rather than being expressed as categorical variables based upon severity e.g. mild, moderate etc). Independent t-tests were therefore used to analyse ODQ, VAS and TSK scores; and Mann-Whitney U for DRAM. Analyses were performed independently for left and right sides because the tasks were asymmetrical. The alpha level was set at 0.05 [23]. %SMVC data was not normally distributed therefore Kruskal-Wallis tests were used. Where differences (*p* < 0.05) were observed Mann-Whitney U tests established pairwise differences between groups (AEP, FP and healthy). To reduce the risk of attaining type 1 errors using multiple Mann-Whitney U tests, a Bonferroni correction was applied and the post hoc significance level set to 0.0167 [23].

## Results

Fifty NSCLBP subjects (23 AEP, 27 FP) and 28 healthy individuals data were analysed. One FP participant failed to complete the patient reported measures. Demographic data is in Table 3. Of note significant differences were observed between groups for gender (AEP 82.6% female, FP 17.4% female). The FP group were significantly heavier (compared to AEP) and taller (compared to AEP and healthy), although BMI was comparable across groups. Participants were matched across groups for age. Back pain location was similar between NSCLBP groups with most subjects reporting central or right-sided symptoms at the time of testing.

**Table 3:** Subject baseline characteristics across groups. (Note: Values are mean (SD) unless otherwise stated)

Variable	AEP	FP	Healthy	Significance
	(*n* = 23)	(*n* = 27)	(*n* = 28)	
Subject Demographics				
Gender				
Males	4 (17.4%)	21 (77.8%)	12 (42.9%)	*p* < 0.001*
Females	19 (82.6%)	6 (22.2%)	16 (57.1%)	
Age (years)	43.7 (11.2)	41.0 (10.0)	38.5 (11.2)	*p* = 0.238
Mass (kg)	68.9 (18.0)	82.5 (14.6)	72.9 (15.2)	*p* = 0.005* (AEP vs. FP)
Height (cm)	164.9 (10.2)	175.9 (8.7)	169.4 (7.3)	*p* < 0.001* (AEP vs. FP/FP vs. H)
BMI (kg/m^2^)	20.8 (4.9)	23.4 (3.5)	21.5 (4.1)	*p* = 0.127
Pain				
Site of Back Pain N(%)				
Right	8 (34.8%)	5 (18.5%)	–	–
Left	2 (8.7%)	3 (11.1%)		
Central	13 (56.4%)	19 (70.4%)		
Time Since Pain Onset N(%)				
3–6 months	2 (8.7%)	8 (29.6%)	–	–
6–12 months	7 (30.4%)	2 (7.4%)	–	–
1–2 years	1 (4.3%)	3 (11.1%)	–	–
2–3 years	0 (0%)	1 (3.7%)	–	–
3–4 years	2 (8.7%)	2 (7.4%)	–	–
4–5 years	3 (13%)	3 (11.1%)	–	–
5–10 years	3 (13%)	4 (14.8%)	–	–
10+ years	5 (21.7%)	4 (14.8%)	–	–
Patient Reported Measures				
	**AEP**	**FP**		
	(*n* = 23)	(*n* = 26)		
ODQ	22.5 (11.6)	21.6 (10.0)	–	*p* = 0.773
DRAM	29.8 (12.5)	22.7 (10.9)	–	p = 0.027*
VAS	4.6 (1.4)	4.5 (1.4)	–	*p* = 0.986
TSK	37.5 (6.8)	37.6 (5.3)	–	*p* = 0.993

Key: FP = Flexion pattern motor control impairment, AEP = Active extension pattern motor control impairment, H = Healthy, BMI = Body Mass Index (mass (kg)/height (m)^2^), kg = kilogrammes, cm = centimetres, *significant difference (*p* < 0.05), ODQ = Oswestry Disability Questionnaire, DRAM = Distress and Risk assessment method, VAS = Visual Analogue Scale, TSK = Tampa Scale of Kinesiophobia

No significant differences between AEP and FP groups in ODQ, VAS, and TSK were revealed. DRAM scores revealed the AEP group displayed significantly greater psychological distress (depressive and somatic distress combined) scores compared to FP (Table 3).

The statistical analysis for the functional tasks are shown in Tables 4 and 5. No significant between group (AEP, FP, Healthy) differences in all muscles were observed during step down, bending to pick up a pen, returning from picking up a pen or sit-to-stand functional tasks. No significant differences were observed between the groups (AEP, FP, Healthy) in the LT muscles during any functional task. Compared to healthy, the AEP group showed significantly greater right-sided sLM activation during step up (*p* = 0.015), reach up (*p* = 0.013) and box replace (*p* = 0.007) tasks. Right EO activity was significantly greater in the AEP group compared to the healthy group during the box lift (*p* = 0.016) task. Left-sided sLM (*p* = 0.009) and TrA/IO (*p* = 0.009) were found to be significantly greater during stand-to-sit in FP compared to the healthy group.

**Table 4:** Results for normalised (%SMVC) amplitude EMG of the right musculature during functional tasks

Task	Muscle	AEP		FP		Healthy		Kruskal-Wallis	Mann Whitney-U Pairwise comparisons (post hoc)
								(*p < 0.05)	(*p < 0.0167)
		Number of trials	Mean (SD)	Number of trials	Mean (SD)	Number of trials	Mean (SD)		
Step Down	TrA/IO	21	57.4 (32.3)	21	62.3 (34.5)	25	52.6 (32.6)	0.576	–
	EO	21	54.4 (26.8)	24	46.2 (23.9)	22	38.8 (21.2)	0.109	–
	sLM	20	33.8 (32.2)	22	24.6 (23.6)	25	16.2 (10.7)	0.136	–
	LT	18	25.8 (20.6)	22	26.2 (17.7)	26	26.9 (24.5)	0.937	–
Step Up	TrA/IO	21	55.1 (31.4)	21	65.0 (39.0)	25	51.1 (33.4)	0.419	–
	EO	21	53.8 (25.0)	24	46.8 (25.0)	22	36.8 (19.7)	0.059	–
	sLM	20	33.8 (33.8)	22	25.0 (24.8)	25	14.6 (9.7)	0.046*	AEP vs H
	LT	18	26.7 (21.0)	22	26.2 (19.2)	26	25.8 (27.9)	0.547	–
Reach Up	TrA/IO	23	50.2 (25.0)	20	63.6 (36.8)	25	50.2 (35.2)	0.284	–
	EO	23	50.7 (23.1)	24	44.2 (23.7)	22	36.7 (19.7)	0.108	–
	sLM	20	26.9 (25.2)	22	19.4 (23.1)	26	12.9 (8.7)	0.039*	AEP vs H
	LT	19	26.7 (26.2)	22	22.7 (16.0)	26	21.1 (16.6)	0.698	–
Pick Up Pen (Bend Down)	TrA/IO	21	54.1 (34.3)	20	64.1 (38.0)	26	50.0 (31.1)	0.391	–
	EO	21	50.2 (23.8)	24	43.9 (25.7)	21	35.0 (17.8)	0.101	–
	sLM	18	28.4 (26.4)	23	20.0 (23.6)	24	16.3 (12.8)	0.132	–
	LT	18	25.6 (21.5)	23	24.0 (18.5)	26	23.6 (19.0)	0.827	–
Pick Up Pen (Return)	TrA/IO	21	54.9 (34.5)	20	62.3 (37.4)	25	48.5 (27.8)	0.45	–
	EO	20	50.3 (26.4)	24	43.2 (25.2)	21	34.5 (17.8)	0.122	–
	sLM	18	29.0 (24.8)	23	20.4 (22.3)	24	16.1 (10.2)	0.089	–
	LT	17	26.9 (21.8)	23	24.1 (17.9)	26	24.2 (23.8)	0.625	–
Stand-to-Sit	TrA/IO	22	59.7 (49.4)	18	68.3 (53.1)	24	44.3 (27.3)	0.266	–
	EO	22	53.6 (25.5)	21	46.2 (24.0)	20	37.8 (20.4)	0.14	–
	sLM	20	50.8 (67.8)	21	31.2 (23.6)	24	19.8 (18.3)	0.054	–
	LT	17	56.3 (79.5)	22	32.1 (17.3)	24	40.8 (48.7)	0.693	–
Sit-to-Stand	TrA/IO	22	58.8 (52.4)	18	55.1 (34.8)	23	42.1 (27.6)	0.381	–
	EO	22	51.5 (24.9)	21	44.6 (23.2)	21	44.0 (37.6)	0.259	–
	sLM	19	44.9 (76.9)	21	23.0 (24.9)	23	12.5 (7.1)	0.254	–
	LT	18	27.7 (22.3)	22	23.5 (15.7)	24	33.5 (47.8)	0.523	–
Box Replace	TrA/IO	23	61.2 (41.8)	20	69.3 (44.2)	25	52.3 (37.5)	0.453	–
	EO	23	55.5 (25.6)	23	44.4 (24.7)	21	37.8 (19.3)	0.062	–
	sLM	19	33.1 (23.3)	23	22.9 (22.5)	25	17.1 (11.1)	0.026*	AEP vs H
	LT	19	33.5 (26.9)	22	24.6 (16.8)	25	27.1 (28.7)	0.486	–
Box Lift	TrA/IO	23	61.3 (42.1)	20	69.7 (44.9)	25	52.0 (38.2)	0.349	–
	EO	23	57.6 (25.6)	23	43.8 (23.4)	21	37.5 (19.1)	0.019	AEP vs H
	sLM	19	32.9 (22.5)	23	24.6 (24.3)	25	18.4 (12.0)	0.051	–
	LT	18	31.9 (25.5)	22	25.3 (16.6)	25	29.0 (32.7)	0.469	–

Key: FP = Flexion pattern motor control impairment, AEP = Active extension pattern motor control impairment, H = Healthy, SD = Standard deviation, * = significant difference (p < 0.05), TrA/IO = Transversus Abdominis / Internal Oblique, EO = External Oblique, sLM = superficial Lumbar Multifidus, LT = Longissimus Thoracis (Erector Spinae)

**Table 5:** Results for normalised (%SMVC) amplitude EMG of the left musculature during functional tasks

Task	Muscle	AEP		FP		Healthy		Kruskal-Wallis	Mann-Whitney U Pairwise Comprisons (Post hoc)
								(*p < 0.05)	(*p < 0.0167)
		Number of trials	Mean (SD)	Number of trials	Mean (SD)	Number of trials	Mean (SD)		
Step Down	TrA/IO	21	78.8 (50.2)	24	74.7 (42.1)	25	76.3 (51.9)	0.948	–
	EO	19	61.5 (39.7)	21	54.3 (22.7)	23	44.7 (23.4)	0.249	–
	sLM	20	19.8 (14.8)	23	19.9 (17.7)	25	13.6 (9.1)	0.287	–
	LT	16	22.7 (12.3)	23	22.7 (15.5)	25	22.8 (12.3)	0.933	–
Step Up	TrA/IO	21	79.4 (55.4)	24	78.9 (43.5)	25	74.7 (58.0)	0.69	–
	EO	19	60.5 (34.3)	21	54.7 (24.0)	23	42.6 (21.9)	0.1	–
	sLM	20	20.2 (16.2)	23	19.7 (18.6)	25	12.7 (8.7)	0.218	–
	LT	16	22.8 (10.9)	23	21.8 (15.3)	25	22.6 (13.3)	0.777	–
Reach Up	TrA/IO	22	70.1 (55.9)	23	71.8 (40.4)	23	58.8 (47.0)	0.229	–
	EO	19	52.6 (34.2)	21	53.2 (24.6)	23	41.9 (20.8)	0.252	–
	sLM	20	19.6 (14.3)	23	19.0 (19.0)	25	13.6 (8.4)	0.38	–
	LT	18	28.6 (17.2)	21	20.2 (15.5)	25	23.4 (14.8)	0.173	–
Pick Up Pen (Bend Down)	TrA/IO	18	77.2 (60.4)	22	73.5 (46.1)	24	76.6 (52.6)	0.957	–
	EO	18	56.6 (33.3)	21	56.8 (32.0)	23	42.2 (20.8)	0.277	–
	sLM	18	25.1 (37.9)	23	18.8 (18.3)	24	14.5 (8.7)	0.812	–
	LT	14	29.6 (36.7)	23	20.5 (15.7)	24	22.4 (13.2)	0.626	–
Pick Up Pen (Return)	TrA/IO	18	75.4 (57.4)	22	74.6 (47.3)	21	64.8 (31.9)	0.869	–
	EO	18	56.9 (3.8)	21	55.4 (31.0)	23	43.9 (22.3)	0.379	–
	sLM	18	25.2 (37.2)	23	18.4 (18.1)	24	14.0 (8.4)	0.624	–
	LT	14	29.2 (35.7)	23	20.9 (16.7)	24	22.1 (12.8)	0.572	–
Stand-to-Sit	TrA/IO	18	60.0 (40.0)	23	76.5 (54.2)	22	39.5 (23.7)	0.02*	FP vs H
	EO	17	58.4 (36.9)	21	55.3 (23.4)	22	40.6 (22.2)	0.094	–
	sLM	19	30.9 (20.8)	22	28.7 (17.1)	22	17.6 (15.4)	0.02*	FP vs H
	LT	15	45.0 (32.0)	22	33.2 (22.4)	23	38.6 (29.9)	0.427	–
Sit-to-Stand	TrA/IO	19	56.0 (35.0)	23	62.6 (44.8)	23	36.7 (21.8)	0.044*	–
	EO	17	52.6 (29.5)	21	53.7 (23.4)	22	39.2 (22.0)	0.115	–
	sLM	17	17.1 (13.5)	22	19.6 (19.8)	23	12.3 (7.9)	0.427	–
	LT	16	29.5 (19.0)	22	22.5 (16.0)	23	28.4 (19.7)	0.301	–
Box Replace	TrA/IO	22	70.5 (39.0)	24	75.8 (41.1)	24	74.1 (63.9)	0.593	–
	EO	19	57.8 (33.1)	21	54.3 (24.8)	23	42.7 (20.7)	0.251	–
	sLM	21	23.4 (14.7)	24	21.0 (18.2)	24	15.1 (9.2)	0.132	–
	LT	17	25.9 (14.6)	22	19.8 (16.2)	24	22.6 (12.9)	0.23	–
Box Lift	TrA/IO	22	72.3 (39.3)	24	76.2 (42.6)	23	74.2 (60.8)	0.66	–
	EO	19	60.0 (35.0)	21	55.4 (25.4)	23	42.5 (21.2)	0.129	–
	sLM	21	23.6 (14.4)	24	20.5 (17.6)	24	15.1 (8.6)	0.123	–
	LT	17	25.5 (13.4)	22	19.4 (15.2)	24	22.6 (13.9)	0.155	–

Key: FP = Flexion pattern motor control impairment, AEP = Active extension pattern motor control impairment, H = Healthy, SD = Standard deviation, * = significant difference (*p* < 0.05), TrA/IO = Transversus Abdominis / Internal Oblique, EO = External Oblique, sLM = superficial Lumbar Multifidus, LT = Longissimus Thoracis (Erector Spinae)

There was a statistically significant difference overall between the groups (AEP, FP, Healthy) during sit-to-stand in the left TrA/IO (*p* = 0.044, *p* < 0.05) however post-hoc testing revealed no significant pairwise between group differences (AEP vs. healthy *p* = 0.056, FP vs. healthy *p* = 0.023).

## Discussion

The purpose of this study was to identify whether differences in muscle activity are task dependent or whether phenotypes of muscle activity can be identified for NSCLBP subgroups irrespective of task performed. Overall, different patterns of muscle activity between tasks and subgroups were observed suggesting potentially individualised trunk muscle responses.

For the AEP group the only differences in muscle activity was when compared with healthy individuals in the right-sided sLM muscles during step up, reach up and box replace tasks (*p* < 0.0167) and in the right EO muscle during the box lift task. For the FP group the only significant differences observed were in the left TrA/IO and sLM muscles during the stand-to-sit task. For all these comparisons muscle activity was greater comparatively in the NSCLBP group compared to the healthy group. For the majority of tasks no significant between group differences in muscle activity were observed. Due to the number of comparisons tested, interpretation of the significant results should be viewed cautiously.

The tasks eliciting significant results for the AEP group, compared to healthy, were step up, reach up and box replace tasks. It could be suggested that these tasks require substantial spinal extension, leading to the AEP group demonstrating greater co-activation of abdominal and extensor musculature during these tasks. Further it could be hypothesised that AEP patients who report pain during extension activities may adopt motor strategies to protect the spine in these movement directions. Trunk musculature hyperactivity in AEP individuals has been previously proposed [1]. In support of clinical observations [1], in nearly all instances the AEP group in this study demonstrated greater overall muscle activation compared to the healthy group (Tables 3 and 4), indicating increased co-activation of the trunk musculature throughout all functional tasks. This was irrespective of the extension or flexion bias to the task. Of note, the AEP group were significantly more psychologically distressed compared to FP (DRAM score: 29.8 vs 22.7 respectively, *p* = 0.027), a factor known to influence NSCLBP [24, 25]. Although this is an interesting observation, the rationale for increased distress in the AEP group is not fully understood and further work evaluating psychological profiles in NSCLBP subgroups is required.

The increased muscle activity demonstrated in the FP compared to the healthy group in the left TrA/IO and sLM during the stand-to-sit activity is less clearly understood. Since no other tasks demonstrated increased TrA/IO activity it is unclear as to whether TrA/IO activity may actually differ between these subgroups. With no consistency in muscle activation between groups noted bilaterally, this further supports the reported inherent wide individual variation in muscle activity [26].

No significant differences were observed between the AEP and FP groups for any muscle group or functional task. Generally the NSCLBP subgroups demonstrated greater muscle activation in each muscle group (compared to the healthy subjects), indicating that co-contraction may be a factor for individuals in pain. Net trunk muscle activity has been previously shown to be increased during the presence of acute pain [26]. This suggests that persistent muscle activation may restrict intervertebral motion as a protective mechanism of the neuromuscular system to increase local spinal stability and thus protect dysfunctional passive spinal structures from pain provocative movement.

Consideration must be made that the tasks were not sufficiently flexion biased to challenge the FP individuals, and nor sufficiently extension biased to challenge the AEP individuals. Additionally, some functional tasks were asymmetrical, (e.g. the box replace task (see Additional file 1)) where the lifting component utilised right trunk rotation). Previous work has demonstrated regional differences in TrA, IO and EO activation during trunk rotation [27] which may have contributed to the unilateral significant differences in muscle activity observed. Also functional tasks can be performed utilising different movement strategies, thus movement variability will be greater than in sitting [6].

Overall, we report differences in muscle activity in MDCS subgroups concurring with previous literature [6–8]. Increased trunk muscle activity is a key feature in the presence of pain [2, 3], however, the study suggests that muscle activity reflects individual patterns of muscle behaviour in NSCLBP cohorts, as well as the healthy subjects. Identifying potential subgroups based on muscle activation responses remains challenging.

### Limitations and future work

Although sEMG is the most commonly used approach for measuring muscle activity in LBP patients [6, 17, 19], there are many confounding variables including ‘cross- talk’ [19] potentially affecting the data.

Although the gender split is reflective of previous subgrouped cohorts [5, 28] (FP: 77.8% male, AEP: 82.6% female) gender could have confounded the results. To exclude this, additional analyses were run using an ANOVA with gender considered as a covariate. Overall the findings were unchanged with this secondary analysis.

Normalizing EMG data from individuals exhibiting pain may lead to an unwillingness or inability to perform maximum exertions [29] hence submaximal contractions were utilised. This is an accepted procedure for normalising data in painful populations [17, 30]. Future work could however seek to explore alternative approaches to normalising EMG data [29] or comparing raw EMG signals. This study presents preliminary work and future, in-depth, analyses of the data, such as time series of normalised EMG, would be beneficial to explore.

Further, with electrode placement at the level of the L5 spinous process for multifidus, EMG recordings may be at risk of being dominated by longissimus and not multifidus activity [31]. Fine wire elecrodes would be required to ensure specificity in future work.

### Clinical implications

When considered in conjunction with work evaluating spinal kinematics in MDCS subgroups [10]. this data can inform rehabilitation approaches. Varying maladaptive movement strategies may predominate in different subgroups (i.e. increased muscle co-activation in AEP; and differences in thoraco-lumbar spinal posture in FP). Thus, targeted interventions should differ between subgroups such as postural re-education for FP to reduce excessive kyphosis and mechanisms to reduce trunk extensor muscle hyperactivity for AEP. However spinal kinematics may be a better discriminator than muscle activity for MDCS subgroups. Further work, for example use of fine wire EMG, ratios of muscle activity and muscle activity *throughout* the functional tasks is required. Further, regional spinal kinematics should be correlated with muscle activity data to establish potential links and treatment targets.

## Conclusions

This study demonstrates differing patterns in trunk muscle activity between subgroups during functional tasks, suggesting potentially individualised trunk muscle responses in the presence of pain. The findings show inconsistencies in muscle activity previously observed in MDCS subgroups [6, 8]. When considered alongside work evaluating MDCS subgroups during functional activity [10] it appears that spinal kinematics may be better at differentiating between clinical (NSCLBP) subgroups, however further work into EMG in NSCLBP subgroups during more demanding functional tasks is warranted to inform targeted interventions for these individuals.

## Supplementary information

**Additional file 1.** Additional Supplementary Material including: Electrode placement; Sub-Maximal Voluntary Contraction Procedures; Functional Task Protocols; and Data Processing of Functional Tasks.

## Data Availability

The datasets used and analysed during this study are available from the corresponding author on reasonable request.
