# Supplementary material for: Investigating differences in trunk muscle activity in non-specific chronic low back pain subgroups and no-low back pain controls during functional tasks: a case-control study
Source: BMC Musculoskelet Disord. 2019 Oct 22;20:459. doi: 10.1186/s12891-019-2843-2 (PMC6805581; doi:10.1186/s12891-019-2843-2)
Supplement: Supplementary file 1 — Additional file 1. Additional Supplementary Material including: Electrode placement; Sub-Maximal Voluntary Contraction Procedures; Functional Task Protocols; and Data Processing of Functional Tasks. [file 12891_2019_2843_MOESM1_ESM.pdf]

Electrode Placement

Electrodes for LT were placed vertically at a point 2 finger widths laterally from the spinous process of L1. For sLM electrodes were placed 2-3cm from the midline (aligned with a line from caudal tip of the PSIS' to the interspace between L1 and L2) at the level of the L5 spinous process[1]. For EO the electrodes were placed slightly inferior to the rib cage along a line connecting the most inferior point of the costal margin and the contralateral pubic tubercle[2-4]. For TrA/IO the electrodes were placed approximately 2cm medially and inferior to the ASIS[5].

References

1. Freriks B, Hermens HJ (1999) [SENIAM 9]: European Recommendations for Surface Electromyography: Results of the SENIAM project. Roessingh Research and Development b.v.
2. Dankaerts W, O'Sullivan P, Burnett A, Straker L (2006) Altered patterns of superficial trunk muscle activation during sitting in nonspecific chronic low back pain patients: importance of subclassification. Spine 31 (17):2017-2023. doi:10.1097/01.brs.0000228728.11076.82
3. Ng JK, Kippers V, Richardson CA (1998) Muscle fibre orientation of abdominal muscles and suggested surface EMG electrode positions. Electromyogr Clin Neurophysiol 38 (1):51-58
4. Dankaerts W, O'Sullivan PB, Burnett AF, Straker LM, Danneels LA (2004) Reliability of EMG measurements for trunk muscles during maximal and sub-maximal voluntary isometric contractions in healthy controls and CLBP patients. Journal of Electromyography and Kinesiology 14 (3):333-342. doi:10.1016/j.jelekin.2003.07.001
5. Marshall P, Murphy B (2003) The validity and reliability of surface EMG to assess the neuromuscular response of the abdominal muscles to rapid limb movement. Journal of Electromyography and Kinesiology 13 (5):477-489

Sub-Maximal Voluntary Contraction Procedures

A crook-lying double leg raise was used to achieve SMVC of the abdominal muscles (knees 90°, hips 45°, feet lifted approximately 1cm off the bed, held for 3 seconds). For the LT and sLM muscles, SMVC values were obtained from a prone lying double knee lift, with the subject lying prone on the plinth (knees 90°, knees lifted approximately 5cm off the bed, held for 3 seconds).

Functional Task Protocols

Sit-to-Stand-to-Sit

For the sit-to-stand task the plinth height was individually standardised to a height where the subjects' hips and knees were resting comfortably at 90 degrees, measured using a goniometer (Lafayette Instrument Co. Ltd., Lafayette, IN) with the thighs well supported on the plinth. Sit to stand was performed from a usual sitting position. The subject was instructed to sit in their usual (unsupported) sitting position on the plinth, wait for 2 seconds in standing, then return to the original position.

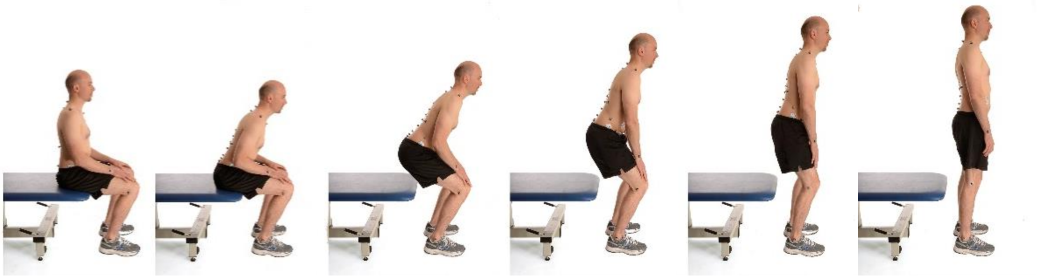

Box

To measure a standardised distance for moving the box during the rotational box task tape was placed at a distance equal to 70% of the total upper limb (UL) length from the midline of the plinth (NB: total upper limb length was measured in cm from the apex of the acromion process to the distal end of the middle phalanx of each hand). For this task the plinth was also set to the height of the individuals' greater trochanter. To perform the task a 2.5kg box was placed over the marked line to the left hand side of the plinth. The subject was instructed to stand with the plinth in front and move the box from left to right (to a position over the line to the right hand side) with the box starting and finishing facing the same direction. No specific directions regarding how to lift were given, however the subject was instructed to stand in a comfortable position and keep their feet stationary throughout the task. At the end of the task the subject returned to their usual standing position.

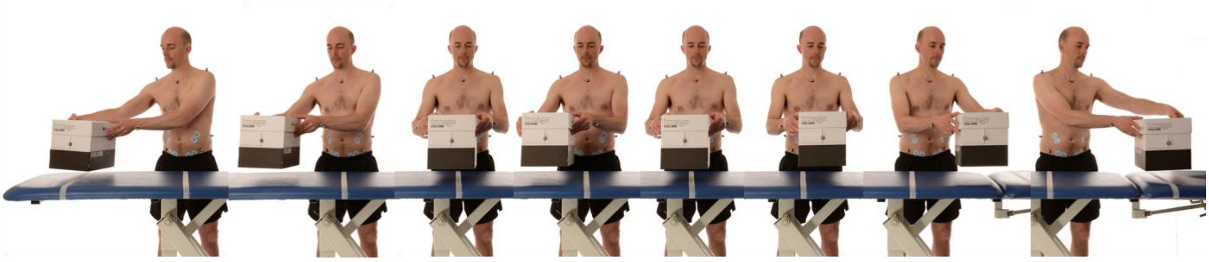

Reaching

The shelf used in the reaching task was set to the height of the ulna styloid process (right upper limb) when the shoulder was in full flexion (fully elevated). The subject stood directly in front of the custom-made shelf, with the shelf base in-line with the midline of the trunk (frontal plane). The subject placed a jar onto the shelf using their right hand, allowed the jar to rest on the shelf for 2 seconds (without releasing from their hand) and returned the jar to the original position. Feet were kept stationary throughout and the subject was instructed to keep their heels on the floor at all times. The subject also kept hold of the jar at all times throughout the task.

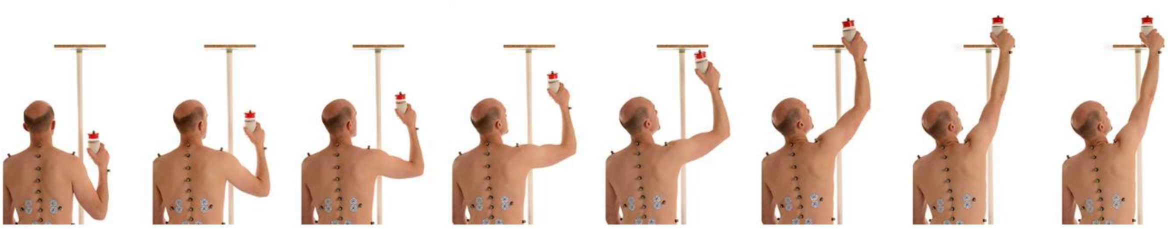

Stepping up and down

Subjects were instructed to stand in front of a 6-inch Reebok® step (Adidas International Trading, Amsterdam, Netherlands), step onto the step (with a self-selected leading-leg), wait in double-stance on top of the step for 2 seconds, and then step down (with a self-selected leading-leg). The subject was instructed that the self-selected leading-leg must remain consistent throughout trials. To ensure data could be analysed effectively in MATLAB the subject was required to wait in their usual standing position following the step down for 2 seconds to enable the end task position to be defined.

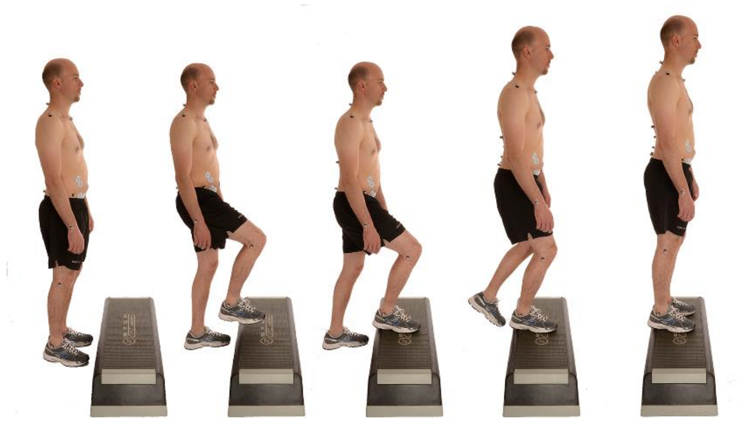

Bending to pick up a pen (and return)

Subjects stood in their usual standing position with a pen (with a marker attached) placed at a point 40cm in front of them on the floor. Subjects were instructed to pick up the pen from the floor and return to their usual standing position. Subjects were encouraged to pick up the pen in whichever way they felt was most natural, however they were instructed to keep their feet stationary throughout the task. Subjects were asked to pick up the pen with their right hand to standardise the movement between subjects.

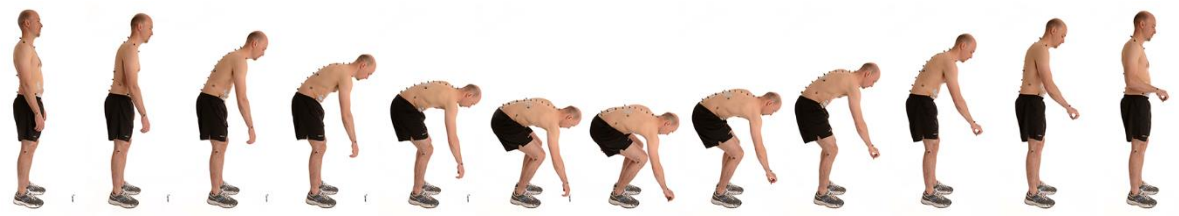

Data Processing of Functional Tasks

The five activities outlined previously (sit-to-stand-to-sit, box lift rotate and replace, bend to pick up pen, step up and down and reaching) were sub-divided into 9 separate tasks in MATLAB as outlined in the table below:

Sit-to-stand-to-sit	Sit-to-Stand
	Stand-to-Sit
Box lift, rotate and replace*	Box Lift
	Box Replace
Bend to pick up pen	Pen Pick Up (Bend Down)
	Pen Pick Up (Return)
Step up and down	Step Up
	Step Down
Reaching	Reach Up
	Reach Down (NB: not included in analysis)

*NB: only the lifting and replacing components of the task were analysed in the current study.
